# 640. A Retrospective Assessment of the Effects of Cefiderocol in Patients with Multidrug-resistant Gram-negative Bacterial Infections

**DOI:** 10.1093/ofid/ofac492.692

**Published:** 2022-12-15

**Authors:** Monirul I Sajib, Roderick Go, Melinda Monteforte

**Affiliations:** Stony Brook University Hospital, West Babylon, New York; Stony Brook University Hospital, West Babylon, New York; Stony Brook University Hospital, West Babylon, New York

## Abstract

**Background:**

Cefiderocol has emerged recently as a new tool for combating multidrug-resistant bacterial infections; however, concerns about its efficacy has approved its FDA approval for the use in person with limited or no treatment options. The aim of our study is to assess the outcomes of patients treated with cefiderocol for such infections.

**Methods:**

Retrospective cohort study which includes all adult patients who received cefiderocol for at least three days from 1 October 2020 to 31 December 2021 at Stony Brook University Hospital. Patients who received multiple courses of cefiderocol during the same hospitalization or are still hospitalized were excluded.

**Results:**

22 patients received cefiderocol and met the inclusion criteria. Average age, Charlson comorbidity index, and length of stay were 63.6 year, 5.4, and 53 days respectively.

6 patients had respiratory tract infections, 3 had bacteremia, 2 had complicated UTI, and 11 had infections in other sites. Bacteria isolates included *Pseudomonas aeruginosa* (14), *Acinetobacter baumannii complex* (8), and *Stenotrophomonas maltophilia* (1).

The 14-day and 28-day all-cause mortality for all patients was 4.5% and 13.6%, respectively. The 14-day and 28-day all-cause mortality for patients with respiratory tract infections was 16.7% and 16.7%, bloodstream infections was 0% and 0%, complicated UTI was 0% and 0% and with infections at other sites was 0% and 15.4%, respectively. These rates are lower compared to CREDIBLE-CR study.

The 28-day all-cause mortality in double gram-negative coverage group (including cefiderocol) was 0% versus 25% in the cefiderocol monotherapy group (p=0.25). The 28-day all-cause mortality in patient group with *Pseudomonas aeruginosa* was 14.3% vs. with *Acinetobacter baumannii complex* was 12.5%. Out of 18 isolates, 13 (72.2%) were susceptible to cefidericol, 1 (5.5%) was intermediate, and 4 (22.2%) were resistant. No carbapenemases were detected in any of the 11 tested clinical isolates of *Pseudomonas aeruginosa*. Treatment failure, defined as requiring change of antibiotic, was 9.1%.

**Conclusion:**

The 14-day and 28-day all-cause mortality for all patients treated with cefiderocol are possibly lower than once thought to be. No significant differences were noted when cefidericol was used as monotherapy versus in combination.

**Disclosures:**

**Roderick Go, DO**, Aptose Biosciences: Stocks/Bonds|Bristol Meyers Squibb: Stocks/Bonds|Cytodyn Inc.: Stocks/Bonds|Scynexis: Grant/Research Support.

